# Endovascular Treatment Effect Diminishes With Increasing Thrombus Perviousness: Pooled Data From 7 Trials on Acute Ischemic Stroke

**DOI:** 10.1161/STROKEAHA.120.033124

**Published:** 2021-07-20

**Authors:** Manon Kappelhof, Manon L. Tolhuisen, Kilian M. Treurniet, Bruna G. Dutra, Heitor Alves, Guang Zhang, Scott Brown, Keith W. Muir, Antoni Dávalos, Yvo B.W.E.M. Roos, Jeffrey L. Saver, Andrew M. Demchuk, Tudor G. Jovin, Serge Bracard, Bruce C.V. Campbell, Aad van der Lugt, Francis Guillemin, Philip White, Michael D. Hill, Diederik W.J. Dippel, Peter J. Mitchell, Mayank Goyal, Henk A. Marquering, Charles B.L.M. Majoie

**Affiliations:** Radiology and Nuclear Medicine (M.K., M.L.T., K.M.T., B.G.D., H.A., G.Z., H.A.M., C.B.L.M.M.); Biomedical Engineering and Physics (M.L.T., B.G.D., H.A., H.A.M.); Neurology (Y.B.W.E.M.R.), Amsterdam University Medical Center, University of Amsterdam, the Netherlands.; Altair Biostatistics, St Louis Park, MN (S. Brown).; Neuroscience & Psychology, University of Glasgow, Queen Elizabeth University Hospital, United Kingdom (K.W.M.).; Neuroscience, Hospital Germans Trias i Pujol, Universitat Autònoma de Barcelona, Spain (A.D.).; Neurology, Comprehensive Stroke Center, David Geffen School of Medicine, University of California, Los Angeles (UCLA) (J.L.S.).; Clinical Neurosciences (A.M.D., M.D.H.), University of Calgary, Alberta, Canada.; Radiology (M.G.), University of Calgary, Alberta, Canada.; Neurology, University of Pittsburgh Medical Center, PA (T.G.J.).; Diagnostic and Interventional Neuroradiology (S. Bracard), University of Lorraine, University Hospital of Nancy, France.; Epidemiology (F.G.), University of Lorraine, University Hospital of Nancy, France.; Medicine and Neurology (B.C.V.C.), Royal Melbourne Hospital, University of Melbourne, Parkville, Australia.; Radiology (P.J.M.), Royal Melbourne Hospital, University of Melbourne, Parkville, Australia.; Radiology and Nuclear Medicine (A.v.d.L.), Erasmus Medical Center, Rotterdam, the Netherlands.; Neurology (D.W.J.D.), Erasmus Medical Center, Rotterdam, the Netherlands.; Institute of Neuroscience, Newcastle University, Newcastle upon Tyne, United Kingdom (P.W.).

**Keywords:** cerebral infarction, decision making, reperfusion, stroke, thrombectomy, tissue-type plasminogen activator

## Abstract

**Background and Purpose:**

Thrombus perviousness estimates residual flow along a thrombus in acute ischemic stroke, based on radiological images, and may influence the benefit of endovascular treatment for acute ischemic stroke. We aimed to investigate potential endovascular treatment (EVT) effect modification by thrombus perviousness.

**Methods:**

We included 443 patients with thin-slice imaging available, out of 1766 patients from the pooled HERMES (Highly Effective Reperfusion Evaluated in Multiple Endovascular Stroke trials) data set of 7 randomized trials on EVT in the early window (most within 8 hours). Control arm patients (n=233) received intravenous alteplase if eligible (212/233; 91%). Intervention arm patients (n=210) received additional EVT (prior alteplase in 178/210; 85%). Perviousness was quantified by thrombus attenuation increase on admission computed tomography angiography compared with noncontrast computed tomography. Multivariable regression analyses were performed including multiplicative interaction terms between thrombus attenuation increase and treatment allocation. In case of significant interaction, subgroup analyses by treatment arm were performed. Our primary outcome was 90-day functional outcome (modified Rankin Scale score), resulting in an adjusted common odds ratio for a one-step shift towards improved outcome. Secondary outcomes were mortality, successful reperfusion (extended Thrombolysis in Cerebral Infarction score, 2B–3), and follow-up infarct volume (in mL).

**Results:**

Increased perviousness was associated with improved functional outcome. After adding a multiplicative term of thrombus attenuation increase and treatment allocation, model fit improved significantly (*P*=0.03), indicating interaction between perviousness and EVT benefit. Control arm patients showed significantly better outcomes with increased perviousness (adjusted common odds ratio, 1.2 [95% CI, 1.1–1.3]). In the EVT arm, no significant association was found (adjusted common odds ratio, 1.0 [95% CI, 0.9–1.1]), and perviousness was not significantly associated with successful reperfusion. Follow-up infarct volume (12% [95% CI, 7.0–17] per 5 Hounsfield units) and chance of mortality (adjusted odds ratio, 0.83 [95% CI, 0.70–0.97]) decreased with higher thrombus attenuation increase in the overall population, without significant treatment interaction.

**Conclusions:**

Our study suggests that the benefit of best medical care including alteplase, compared with additional EVT, increases in patients with more pervious thrombi.

## Abstract 2

Supplemental Digital Content is available in the text.

In acute ischemic stroke, thrombi are often assumed to completely occlude vessels, blocking all flow like a cork on a bottle of wine. However, some thrombi are permeable, allowing for residual flow into and through them.^1–3^ Thrombus perviousness estimates residual flow through thrombi based on routine noncontrast CT (NCCT) and single-phase CT angiography (CTA).^4,5^ Perviousness was associated with improved outcomes and recanalization rates after intravenous alteplase.^4^ For endovascular treatment (EVT) however, reported effects of perviousness vary considerably.^6,7^

The current standard treatment of acute ischemic stroke due to anterior circulation large vessel occlusions consists of alteplase if patients are eligible, followed by EVT.^8,9^ Although EVT has greatly improved outcomes, even after EVT more than half of patients remain permanently disabled or die after their stroke.^9^ The added benefit of intravenous alteplase is investigated in several recently completed and ongoing randomized trials.^10^ Alteplase benefit may vary case-by-case: large proximal occlusions are known to show little benefit,^11^ while 10% of EVT-eligible patients recanalize with alteplase before initiation of EVT.^12^ Selecting the treatment with the highest chance of benefit (alteplase, EVT, neither, or both) is, therefore, expedient and could enable a more targeted and effective use of treatment modalities.

Currently, no radiological thrombus characteristics other than occlusion location are used in acute ischemic stroke decision making.^8^ The possible association of thrombus perviousness with alteplase treatment success, either alone or combined with EVT, could play a role in improving stroke treatment selection.^4,5^ To assess the role of perviousness, a large data set is needed, enabling separate analysis of treatment approaches. We aimed to investigate the effect of thrombus perviousness on EVT results in the HERMES trial (Highly Effective Reperfusion Evaluated in Multiple Endovascular Stroke) pooled data set of 7 large randomized trials on EVT for stroke.^9,13–19^

## Methods

### Patients

The HERMES collaboration pooled individual patient data from 7 randomized controlled trials on EVT in acute ischemic stroke of the anterior circulation,^9,13–19^ totaling 1766 patients included between December 2010 and December 2014. Inclusion criteria of the individual trials were reported previously.^13–19^ Pooling protocol, study selection, risk of bias, individual patient data acquisition, and data checks are described in the original pooling report.^9^ Control arm patients received best medical care, including alteplase if eligible. Intervention arm consisted of additional EVT.

**Figure 1.**
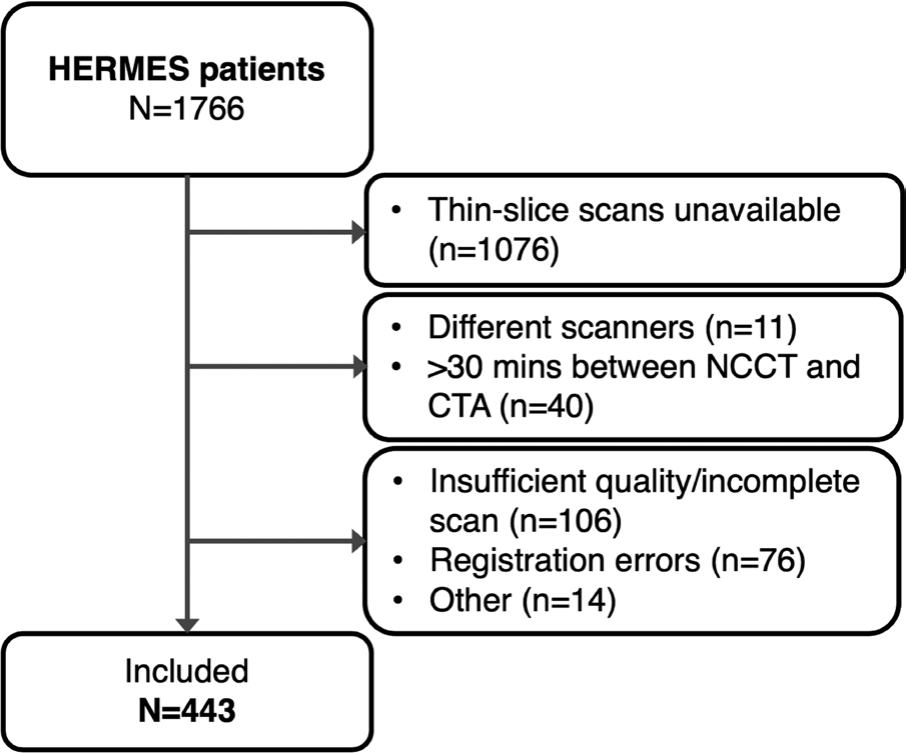
**Patient inclusion flowchart.** Other includes thrombus too close to bone (n=6), only partial occlusion (n=4), too narrow thrombus (n=3), bilateral middle cerebral artery thrombus (n=1). CTA indicates computed tomographic angiography; HERMES, Highly Effective Reperfusion Evaluated in Multiple Endovascular Stroke; and NCCT, noncontrast computed tomography.

Patients were included in the current study if thin-slice (≤2.5 mm) NCCT and CT angiography (CTA) images were available (n=690). We excluded patients if NCCT and CTA images were on different scanners (n=11), or >30 minutes apart (n=40). We excluded patients with scans that did not cover the intracranial area of interest (n=33), were of insufficient quality (n=93; beam hardening: n=50, movement artifacts: n=32, contrast present on NCCT: n=6, scatter artifacts: n=4, and venous phase CTA: n=1), or with incorrigible registration errors (n=76). Thrombi located too close to bone (n=6), partial occlusions (n=4), narrow and distal thrombi (n=3), and bilateral middle cerebral artery thrombi (n=1) were excluded. The remaining 443 patients were included in the final analysis (Figure 1). The HERMES data used for this study are available via the VISTA-Endovascular repository. We followed the PRISMA-IPD guideline for study execution and reporting (Methods in the Data Supplement).

### Thrombus Perviousness Measurements

NCCT and CTA images were coregistered using rigid registration with Elastix.^20^ Thrombus perviousness was quantified by measuring thrombus attenuation increase (TAI) between NCCT and CTA images as described previously.^4^ For image selection and ROI placement, open-source software ITK-SNAP was used.^21^

**Figure 2.**
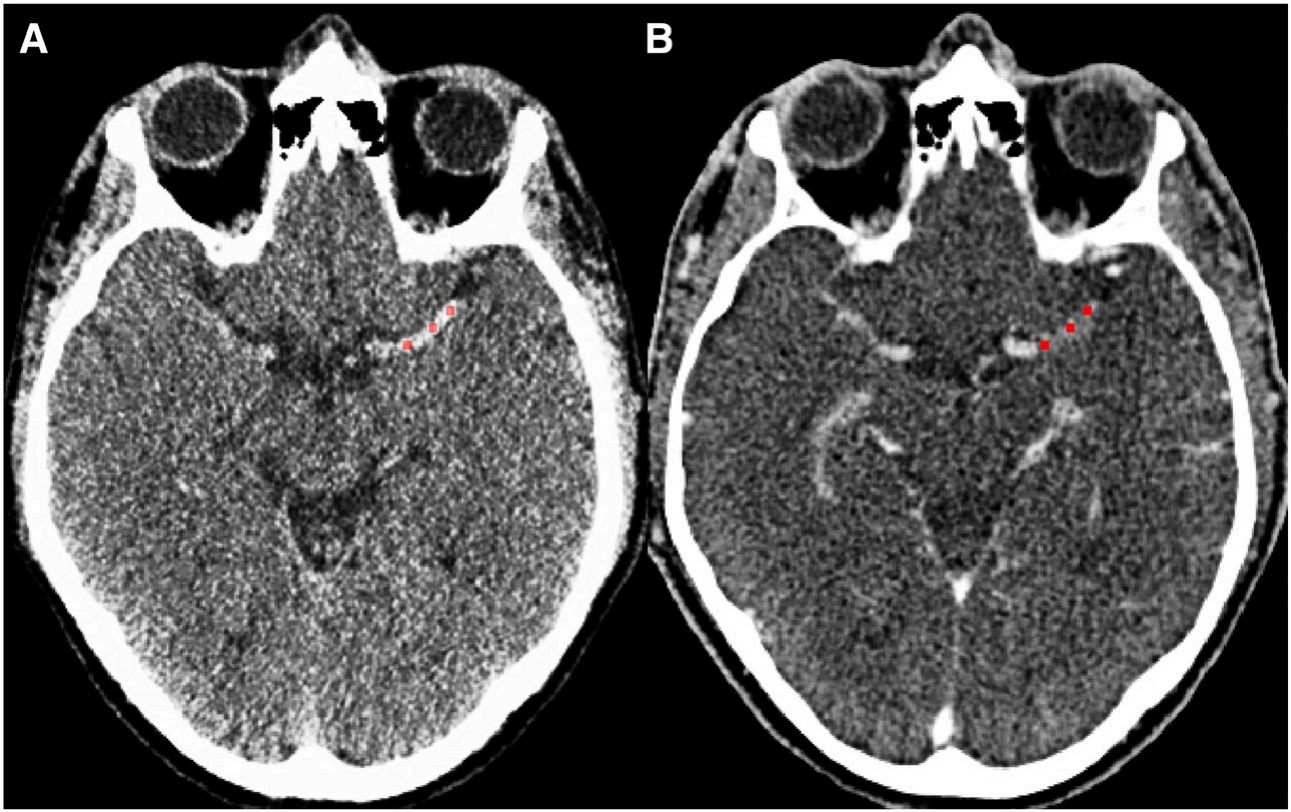
**Thrombus attenuation increase measurement.** Three regions of interest (red) are placed in a left middle cerebral artery thrombus on noncontrast computed tomography (CT; **A**) and CT angiography (**B**). To calculate thrombus attenuation increase as a measure for perviousness, the density (in Hounsfield Units) in each region of interest is measured. Subsequently, the average noncontrast CT attenuation value is deducted from the average CT angiography value.

Thrombus attenuation was measured manually by placing 3 spherical ROIs with a radius of one millimeter in the proximal, middle, and distal parts of the thrombus (Figure 2). The average density (ρ in Hounsfield units [HU]) of all ROIs was calculated for NCCT and CTA (ρ_NCCT_ and ρ_CTA_). TAI was calculated according to: TAI=ρ_CTA_−ρ_NCCT_.^5^

Measurements were performed by one of 3 trained raters (Drs Kappelhof, Dutra, and Alves).^22^ Raters were blinded for occlusion location, clinical information, and measured attenuation values. Training was done with 25 cases from the MR CLEAN Registry.^23^ Scan acquisition characteristics including NCCT and CTA slice thickness difference (CTA slice thickness minus NCCT slice thickness),^24^ scanner brand, and scanner kVp were recorded for all included patients.

### Outcome Measures

Our primary outcome was functional outcome (ordinal modified Rankin Scale [mRS]) at 90 days. The mRS score ranges from 0 to 6, with 0 indicating complete functional independence, and 6 indicating death. Secondary outcome measures were dichotomized functional outcome (mRS score, 0–2 versus 3–6 indicating functional independence; mRS score, 5–6 versus 0–4 indicating poor outcome), mortality, successful reperfusion (extended Thrombolysis in Cerebral Infarction score grade, 2B–3) after EVT (intervention arm patients only), and follow-up infarct volume (FIV) in mL as measured on follow-up NCCT.^25^

### Statistical Analysis

Statistical analyses were prespecified in a statistical analysis plan (Methods in the Data Supplement). Baseline clinical, imaging, and follow-up variables were compared with the overall HERMES population and between every quartile of TAI. One-way ANOVA was used to assess differences in normally distributed numerical variables, Kruskal-Wallis and Mann-Whitney *U* tests for non-normal numerical variables, and Fisher exact tests for categorical variables.

TAI outliers (n=11) were identified with the outlier labeling rule with a multiplier of 2.2, marking them as outliers if their difference with the median was ≥2.2× the interquartile range (IQR).^26^ For 10 patients (2%), one marker was adjusted due to erroneous placement outside the vessel or thrombus. All remaining outliers (n=4, 1%) were attributable to short pervious thrombi, random noise, or slice thickness differences between NCCT and CTA, and were included in the final analysis.

Univariable and multivariable ordinal logistic regression were used in the primary outcome variable analysis (mRS shift). Univariable and multivariable binary logistic regression was used for dichotomized outcome variables. Associations between TAI and FIV were tested with linear regression. Because FIV showed a right-skewed distribution, log-transformation was performed. Associations between TAI and outcomes are reported per 5 HU.

Regression analyses were adjusted for age, baseline National Institutes of Health Stroke Scale score, intravenous alteplase, occlusion location, diabetes, stroke onset to randomization time, slice thickness difference between NCCT and CTA, and included random effects for allocated study and scanner brand. Unadjusted and adjusted (common) odds ratios (u[c]OR and a[c]OR) were reported with 95% CI. We added an interaction term between TAI and allocated treatment in separate, subsequent models. For successful reperfusion as outcome measure, an additional regression model included an interaction term between TAI and alteplase treatment. If treatment interaction was significant, subgroup analyses were performed. Exploratory subgroup analyses were performed using unadjusted and adjusted ordinal logistic regression for EVT effect per quartile of TAI, for the primary outcome only.

Statistical analyses were performed with R version 3.5.2 (R Foundation for Statistical Computing, Vienna, Austria). The significance level was set at *P*<0.05.

## Results

Median age was 67 years (IQR, 59–76), median baseline National Institutes of Health Stroke Scale score was 18 (interquartile range, 14–21), and 53% of patients were male (Table 1). In comparison to the overall HERMES population, mRS scores, occurrence of sICH, and FIV were slightly higher. Intravenous alteplase was administered to 91% of the patients in the control arm and 85% of the patients in the intervention arm.

**Table 1.**
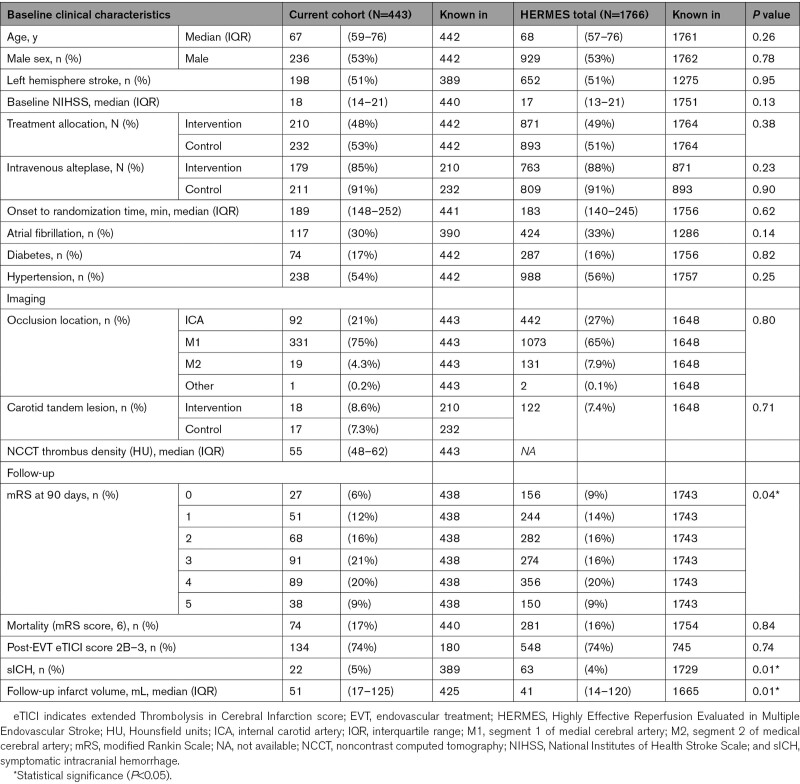
Baseline and Follow-Up Characteristics

TAI showed a slightly right-skewed distribution with a mean of 4.5 HU (SD, 12.7) and median 3.2 HU (IQR, −4.3 to 11.4; Figure I in the Data Supplement). Baseline characteristics did not significantly differ between TAI quartiles (Table I in the Data Supplement). TAI values did not differ between patients with and without extracranial carotid tandem lesions (*P*=0.47). Scan acquisition details are discussed in Results in the Data Supplement.

### Primary Outcome

**Figure 3.**
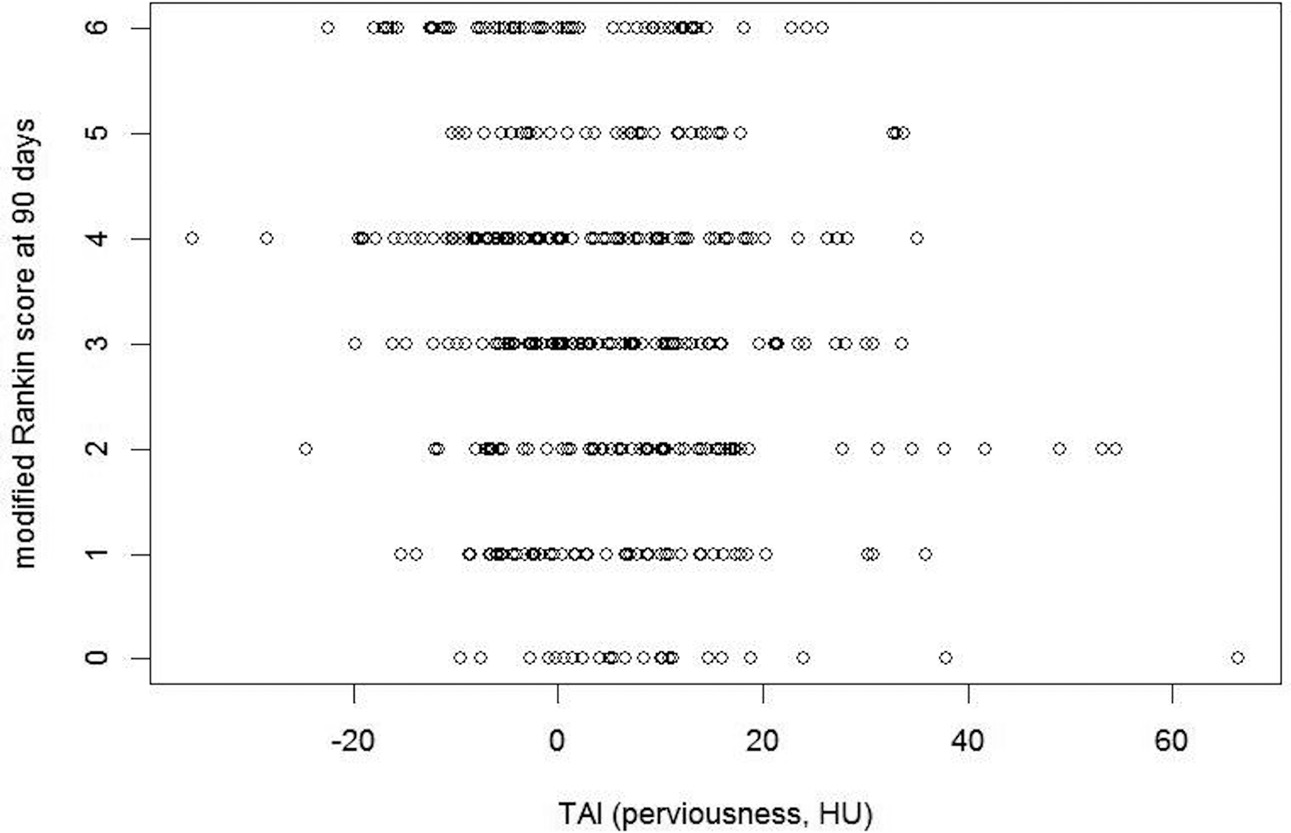
Thrombus attenuation increase (TAI) values varied per modified Rankin Scale (mRS) score (*P*<0.001).

Ninety-day mRS was available for 438 of 443 patients. Higher TAI corresponded to lower mRS (*P*<0.01; Figure 3). There was significant interaction between TAI and allocated treatment (*P* for interaction, 0.03; Table 2). In the control arm, TAI was associated with improved outcomes (acOR, 1.22 [95% CI, 1.11–1.33]). In the intervention arm, no significant effect was found (acOR, 0.99 [95% CI, 0.88–1.11]). These results were consistent in an exploratory analysis of alteplase-treated patients (390/443, 88%; Table III in the Data Supplement). Analysis per TAI quartile showed a nonsignificant benefit of EVT in the highest quartile (Table IV in the Data Supplement).

**Table 2.**
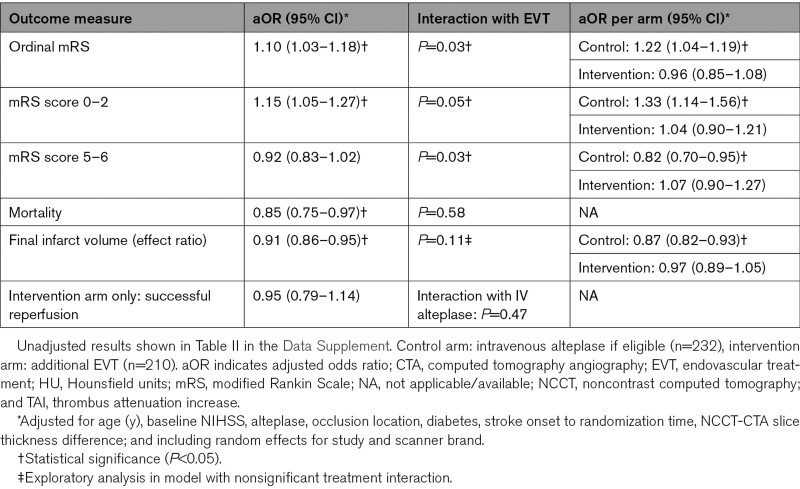
aOR for the Effect of TAI (per 5 HU) on Outcomes

### Secondary Outcomes

#### Functional Independence

In the control arm, patients with 90-day mRS score of 0 to 2 (n=59/231; 26%) had more pervious thrombi than patients with mRS score of 3 to 6 (median, 8.5 [IQR, 1.1–17.4]) versus 1.2 [IQR, −4.9 to 9.4], *P*≤0.01, respectively). In the intervention arm, TAI values did not significantly differ between patients with mRS score of 0 to 2 and mRS score of 3 to 6 (Figure II in the Data Supplement).

**Figure 4.**
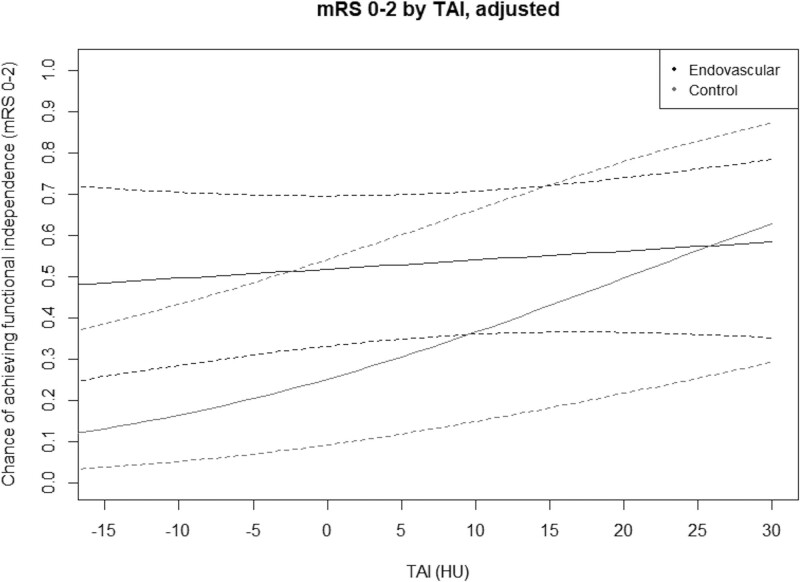
**Probability of functional independence (90-d modified Rankin Scale [mRS] score, 0–2) vs thrombus attenuation increase (TAI).** Results are adjusted for prespecified variables age, baseline National Institutes of Health Stroke Scale, alteplase, occlusion location, diabetes, stroke onset to randomization time, and noncontrast computed tomography-computed tomography angiography slice thickness difference. Control arm: best medical care (intravenous alteplase if eligible; n=232). Intervention arm: endovascular treatment in addition to best medical care (n=210).

Interaction between TAI and allocated treatment was significant (*P*=0.046). Subgroup analysis showed a significantly positive association between TAI and functional independence in the control arm, but not in the intervention arm (Table 2). Figure 4 shows the adjusted probability of functional independence plotted against TAI, for intervention and control arm patients. Treatment effect of EVT in addition to best medical care (alteplase if eligible) decreases with higher TAI, although there is no point where the CIs are separated. Unadjusted results are presented in Figure III in the Data Supplement.

### mRS Score of 5 to 6 and Mortality

Higher TAI was associated with a lower chance of poor outcome in the control arm (*P* for interaction 0.03; Table 2). Greater TAI also corresponded to a smaller chance of mortality in the overall population (aOR, 0.85 [95% CI, 0.75–0.97]), but treatment interaction was not significant (*P*=0.58).

### Successful Endovascular Reperfusion

eTICI scores were available for 180/210 intervention arm patients (86%). Successful reperfusion was reached in 134/180 cases (74%). We found no significant effect of TAI on reperfusion (Table 2). Interaction between TAI and alteplase was not significant for successful reperfusion (*P*=0.47)—although only 31 patients (15%) in the intervention arm did not receive intravenous alteplase. Post-EVT eTICI scores were known in 26 of them (12% of all intervention arm patients).

### Follow-Up Infarct Volume

A 5 HU increase in TAI was associated with a 9% decrease in FIV (95% CI, 14%–5%). Interaction between TAI and treatment arm was not significant (*P*=0.11; Table 2; Figure IV in the Data Supplement).

## Discussion

The benefit of EVT as an addition to alteplase diminishes in patients with more pervious thrombi, due to improved outcomes with increasing perviousness in patients receiving alteplase alone. Increased thrombus perviousness was associated with improved functional outcome in HERMES control arm patients, who, in a high proportion, were treated with intravenous alteplase. No significant effect of perviousness on functional outcome was found in patients included in the intervention arm, who received EVT in addition to best medical care. Results of associations with secondary outcomes were similar. No value for perviousness was observed where better outcomes were associated with withholding EVT, though the association with functional outcome was more pronounced in the higher perviousness quartiles.

Our findings generally agree with previous studies showing increased recanalization rates and improved outcomes in patients with more pervious thrombi after intravenous alteplase treatment.^4,5,7,27–29^ These studies, however, compared intravenous alteplase to conservative treatment only^4^ or found no interaction with EVT, possibly due to lower patient numbers.^5^ Improved target vessel recanalization for pervious thrombi seems to lead to improved outcomes after intravenous alteplase,^28^ on average leaving less opportunity for EVT to give additional benefit. On the association between perviousness and EVT, results vary: in contrast to our results, a recent study in EVT-treated patients reported a significantly positive association between perviousness and functional outcome in the adjusted analyses.^7^ Perviousness was reported to be associated with higher chances of first-pass success of aspiration thrombectomy.^30^

Previous studies have dichotomized TAI, to classify thrombi into pervious and nonpervious.^4,5^ The optimal cutoff value varied between studies and data sets. However, these values were subsequently used by other study groups without testing validity in their data.^31^ Therefore, and since dichotomization was not necessary for our research question, we did not dichotomize TAI. Following this reasoning, the TAI values in the per-quartile analysis should be interpreted with care. Though interesting, the subgroup analysis is not meaningfully powered with ≈110 patients per group: TAI cutoff values may not be applicable to other data sets.

The observed effect of perviousness may be partly explained by thrombus histopathology. A study on thrombi retrieved during EVT showed a positive correlation between perviousness and fibrin/platelet fractions.^31^ However, conflicting results exist: a recent study reported opposite results.^32^ RBC-rich thrombi were found to be more responsive to thrombolytic therapy.^33^ In relation to EVT, fibrin-rich thrombi were associated with longer intervention times and a higher chance of secondary embolisms.^34^ Thrombus perviousness may also be associated with stroke cause, though published results on this topic are inconsistent.^35,36^

Interestingly, we observed some negative values for TAI. Slice thickness differences between NCCT and CTA could affect density measurements due to partial volume effect.^24^ Minor coregistration errors could minimally offset marker placement in NCCT or CTA. Random (Gaussian) noise could cause affect HU values on either NCCT or CTA. These factors unlikely affect TAI measurement validity: random noise and small coregistration flaws are likely random, we adjusted for slice thickness differences, and our results are reproduceable in studies using the same methods.^4,7^ An effect of differences in scanner voltage on HU values was not supported by our data.

### Limitations

We excluded patients due to unavailable thin-slice imaging. Alternative methods like qualitative assessment of residual flow is possible using thick-slice NCCT but less precise.^24^ Almost all centers acquire thin-slice imaging initially but do not store such images: perviousness could be measured for many more patients if thin-slice imaging is preserved, and a practical, fast, (automatic) measurement method is available.

In our sample, we had no information on alteplase administration before scan acquisition. In patients receiving thrombolysis before transfer to an EVT-performing center, alteplase may have been started before scan acquisition, already exposing some thrombi to alteplase. The effects of alteplase administration on thrombus perviousness are currently unknown. Patients may have recanalized and recovered after alteplase and before randomization, leading to a possible inclusion bias, though start of EVT was not delayed by waiting for the effect of alteplase (except for REVASCAT, n=206/1766).^17^ These patients may have had more pervious thrombi; thereby the current study may under-represent patients with very pervious thrombi and underestimate the effect of perviousness. Likewise, we did not know the time from alteplase administration to CTA acquisition. Thrombus perviousness might increase if alteplase has had more time to act on the thrombus.

Our data consisted of pooled data from multiple trials. We cannot exclude an uneven distribution of the number of included patients per trial. However, since part of the imaging was requested from trials individually, and scanning protocols were heterogeneous, we are reasonably sure that patients from each trial were included. In addition, we included a random effect for allocated study in all adjusted regression analyses.

Technical aspects of image acquisition can influence TAI measurements. Slice thickness of the images we used varied from 0.6 to 2.5 mm. Thicker slices give a lower measured density on NCCT due to volume averaging with surrounding brain tissue.^24^ In addition, CTA scan timing can influence TAI, by slightly differing scanning phase. Dynamic or multiphase CTA incorporates the time dimension by making CTA scans at multiple time points after contrast injection, which can avoid the issue of scanning too early after contrast bolus.^37^

Difficult delineation of thrombi can hamper measurements. The distal thrombus border is hard to discern in case of poor collaterals on CTA and an isodense thrombus on NCCT. However, this occurred only in nine patients in our data set. Measurement in dynamic CTA may show the distal thrombus border more accurately in patients with poor collateral flow.^37^

Finally, it is important to note that although we found significant treatment interaction and decreasing additional benefit of EVT with increasing perviousness, we did not find a TAI value where control arm patients did significantly better than intervention arm patients. Thus, our results do not provide definitive evidence to withhold treatment based on a certain value of perviousness.

Further research could focus on combinations of thrombus characteristics, like perviousness, length, and location, in relation to alteplase and EVT response. Small-volume, short, pervious, distal M2-thrombi may benefit from alteplase, whereas proximal, large-volume, long, impervious ICA terminus occlusions may not. Larger thrombi may respond better to intravenous tenecteplase.^38^ Additionally, the effect of perviousness on outcomes of combined alteplase and EVT versus direct EVT only, or on EVT device outcomes could be studied further. In the future, thrombus perviousness may support patient selection for alteplase alone, combined alteplase and EVT, or EVT alone, and support endovascular treatment modality choice.

### Conclusions

In patients treated in the control arm of HERMES, of whom most were treated with alteplase, increased thrombus perviousness was associated with improved functional outcome, decreased mortality, and reduced infarct volume. We found no significant association among patients allocated to the EVT-arm. The benefit of EVT as an addition to best medical care including alteplase diminishes in patients with more pervious thrombi, due to improved outcomes with increasing perviousness in patients receiving alteplase alone—though no value for perviousness was observed where withholding EVT was associated with better outcomes.

## Acknowledgments

We thank the HERMES (Highly Effective Reperfusion Evaluated in Multiple Endovascular Stroke) collaborators.

## Sources of Funding

The HERMES (Highly Effective Reperfusion Evaluated in Multiple Endovascular Stroke) collaboration was funded by a grant to the University of Calgary from Medtronic LLC.

## Disclosures

Dr Kappelhof reports MRCLEAN-NOIV coordinator. Dr Brown reports personal (University of Calgary, Medtronic). Dr Muir reports personal (Boehringer Ingelheim, Bayer, Daiichi Sankyo, Biogen, ReNeuron). Drs Roos and Marquering reports shareholder (Nico-Lab). Dr Saver reports personal (Medtronic). Dr Demchuk reports personal (Medtronic, Boehringer Ingelheim), patent (Circle NVI). Dr Jovin received personal (Ceronovus, Codman Neurovascular, Neuravi, Stryker, Fundacio Ictus), grants (Stryker Neurovascular, GE Healthcare), shares (Silk Road, Anaconda, Route 92, Blockade), other (Anaconda, Route92, Vizai, FreeOx, Corindus, Methinks, Contego Medical). Dr Campbell reports the following institutional (National Health and Medical Research Council, Royal Australasian College of Physicians, Royal Melbourne Hospital Foundation, National Heart Foundation, National Stroke Foundation of Australia, Medtronic). Dr van der Lugt received grants (GE Healthcare, Siemens Engineers, Thrombolytics Science Inc, Ceronovus, Penumbra, Stryker, Medtronic, AngioCare BV, Covidien/EV3, Medac GmbH/Lamepro, Dutch Heart Foundation, Thrombolytic Science Inc). Dr White reports the following institutional (Stryker, Penumbra, Medtronic), personal (Microvention). Dr Hill reports the following institutional (NoNO Inc, Boehringer Ingelheim), patent (Systems and Methods for Assisting in Decision-Making and Triaging for Acute Stroke Patients), director (Canadian Neuroscience Federation, Canadian Stroke Consortium, Circle Neurovascular). Dr Dippel reports the following institutional (Dutch Heart Foundation, AngioCare BV, Covidien/EV3, Medac GmbH/Lamepro, Penumbra, Top Medical/Concentric, Stryker, Ceronovus, Brain Foundation Netherlands, the Netherlands Organisation for Health Research and Development, Health Holland Top Sector Life Sciences and Health, Medtronic). Dr Mitchell reports the following institutional (Medtronic, Stryker, Codman, Johnson & Johnson). Dr Goyal reports personal (Medtronic, Stryker, Microvention), patent (Systems of acute stroke diagnosis). Dr Majoie received grants (CardioVasculair Onderzoek Nederland/Dutch Heart Foundation, European Commission, Dutch Health Evaluation Program, Stryker, Toegepast Wetenschappelijk Instituut voor Neuromodulatie [TWIN] Foundation).

## Supplemental Materials

Expanded Methods (Statistical Analysis Plan)

Expanded Results (Scan Acquisition Effects)

Online Tables I–IV

Online Figures I–IV

## Supplementary Material


