# Supplementary material for: Endovascular Treatment Effect Diminishes With Increasing Thrombus Perviousness: Pooled Data From 7 Trials on Acute Ischemic Stroke
Source: Stroke. 2021 Jul 20;52(11):3633–41. doi: 10.1161/STROKEAHA.120.033124 (PMC8547583; doi:10.1161/STROKEAHA.120.033124)
Supplement: Supplementary file 1 [file str-52-3633-s001.pdf]

SUPPLEMENTAL MATERIAL

Contents

Supplemental Methods		**2**
	Statistical Analysis Plan	*2*
	PRISMA-IPD Checklist	*5*
	PRISMA-IPD Flowchart	*10*
Supplemental Results		**11**
	Supplemental Results Table 1: Imaging acquisition details	*11*
	Supplemental Results Figure 1: Effects of acquisition details on TAI	*12*
Supplemental Tables		**13**
	Supplemental Table I: Baseline, imaging, outcome data per TAI quartile	*13*
	Supplemental Table II: Unadjusted effect estimates	*15*
	Supplemental Table III: Analysis in intravenous alteplase-treated patients	*16*
	Supplemental Table IV: Effect estimates per TAI quartile for mRS	*16*
Supplemental Figures		
	Supplemental Figure I: TAI distribution	***17***
	Supplemental Figure II: TAI for 90-day mRS 0-2 and 3-6	*17*
	Supplemental Figure III: Unadjusted probability of mRS 0-2	*18*
	Supplemental Figure IV: FIV and TAI per treatment allocation	*19*

SUPPLEMENTAL METHODS

Statistical Analysis Plan

Thrombus perviousness and effect of endovascular treatment in acute ischemic stroke: analysis of the HERMES collaboration data.

We aim to determine the effect of thrombus perviousness on functional outcome in acute ischemic stroke and its modification on the effect of endovascular treatment (EVT). Thrombus perviousness is represented by thrombus attenuation increase (TAI) in Hounsfield Units (HU).

I. Primary study objectives
   a. Determine the association between TAI and functional outcome.
   b. Determine the modification of endovascular treatment effect by TAI with ordinal mRS as outcome measure.

II. Secondary objectives
   a. Determine the association between TAI and dichotomized functional outcome (mRS 0-2 vs. 3-6; 0-4 vs. 5-6; 0-5 vs. 6).
   b. Determine the modification of EVT effect by TAI, with dichotomized functional outcome as outcome measure.
   c. Determine the association between TAI and post-EVT reperfusion (eTICI 2B-3).
   d. Determine the modification of intravenous recombinant tissue plasminogen activator (IV rtPA) effect by TAI, with post-EVT reperfusion as outcome measure.
   e. Determine the association between TAI and final infarct volume.
   f. Determine the modification of EVT effect by TAI, with final infarct volume as outcome measure.

III. Imaging Variables
   a. TAI in HU (TAI=$\rho_{\text{thrombus}}^{\text{CTA}}$ - $\rho_{\text{thrombus}}^{\text{NCCT}}$)(by substudy research group)
   b. Occlusion location (by substudy research group)
   c. Laterality defined as left versus right hemisphere stroke
   d. Final infarct volume in ml

IV. Primary Outcome
   a. Functional outcome according to the modified Rankin Scale (mRS) at 90 days

V. Secondary outcomes
   a. Dichotomized functional outcome
      - 0-2 versus 3-6 (‘functional independence)
      - 0-4 versus 5-6 (‘poor outcome’)
      - 0-5 versus 6 (mortality)
   b. Reperfusion (eTICI 2B-3 versus 0-2A)
   c. Final infarct volume in ml

Statistical analyses

I. Reporting of baseline and follow-up characteristics

Medians and Interquartile Ranges (IQR) will be reported for all continuous variables. Numbers and percentages (n(%)) will be reported for all categorical and dichotomous variables. All baseline characteristics will be shown for the overall patient group with TAI measurements available, and per quartile of TAI, and compared to the overall HERMES patient cohort; see Manuscript Table 1 and Supplementary Table 1.

Baseline characteristics:

- Age (years; continuous)
- Sex (dichotomous)
- Affected hemisphere (dichotomous)
- NIHSS at baseline (ordinal)
- Treatment allocation (intervention/control arm; dichotomous)
- IV rtPA treatment within intervention and control arm (nominal)
- Time from onset to randomization (in minutes; continuous)
- Atrial fibrillation (dichotomous)
- Diabetes mellitus (dichotomous)
- Hypertension (dichotomous)

Imaging characteristics (by substudy researcher group):

- TAI (continuous)
- Occlusion location (ICA/M1/M2/Other[A1]; nominal)
- Thrombus density on NCCT (continuous)
- Thrombus length (continuous)
- NCCT scanner KVP (continuous)
- CTA scanner KVP (continuous)
- NCCT slice thickness (continuous)
- CTA slice thickness (continuous)
- Scanner brand (categorical)

Follow-up characteristics:

- Ordinal functional outcome (mRS at 90 days; ordinal)
- Mortality (mRS 0-5 vs. 6; dichotomous)
- sICH (as defined by each trial; dichotomous)
- Final infarct volume (ml, continuous; by substudy research group)

II. Analysis of primary and secondary study objectives

For all primary and secondary study objectives, regression analyses will be performed. TAI grouped per 5 HU is used as the independent variable. The primary/secondary outcome of interest is used as the dependent variable.

Regression analyses/mixed effects models

For every objective, four regression analyses will be performed: adjusted and unadjusted, with and without interaction term.

Model	Random effect	Adjusted for	Interaction term
1a	Study	-	

1b	Study		TAI*treatment
2a	Study + scanner brand	NCCT-CTA slice thickness difference + pre-specified variables[#]	
2b	Study + scanner brand	NCCT-CTA slice thickness difference + pre-specified variables[#]	TAI*treatment

In the 'b' models, we include an interaction term of TAI*treatment allocation (EVT) for all Study Objectives except for Secondary Objective d.
For Secondary Objectives d, the tested interaction is between TAI and IV rtPA treatment, for all outcome measures.

The analyses will be adjusted for the following pre-specified variables: treatment allocation, IVT yes/no, age, occlusion site, atrial fibrillation, diabetes, and time from stroke onset to IVT, NCCT-CTA slice thickness difference. Random effects will be included for study in Model 1a and 1b, and study and scanner brand in Model 2a and 2b.

Unadjusted odds ratios (uOR; Model 1a and 1b) and adjusted odds ratios (aOR; Model 2a and 2b) with confidence intervals and corresponding p-values for interaction terms will be presented in Manuscript Table 2 and Supplementary Table 2 and 3.

In case of a significant interaction term, subgroup analyses will be performed to acquire separate uORs and aORs for the intervention group and control group patients, also to be presented in Manuscript Table 2 and Supplementary Table A2.

A. Primary study objective: mRS at 90 days (mRS shift analysis)

- Ordinal logistic regression/mixed effect models according to Model 1 and 2.

B. Secondary study objectives

- Dichotomized mRS: logistic regression/mixed effects models according to Model 1+2; for both functional independence and poor outcome (0-2 vs. 3-6 and 0-4 vs. 5-6).
- Mortality (mRS 0-5 vs. 6): logistic regression/mixed effects Model 1+2.
- Successful reperfusion: logistic regression/mixed effects Model 1+2.
- Final infarct volume: linear regression/mixed effects Model 1+2.

<u>Figures</u>
Primary study objective (ordinal mRS):
Scatter plots and boxplots. Regression results for adjusted/unadjusted analyses, and treatment subgroups with plotted confidence intervals.

Secondary study objectives:
For dichotomized mRS 0-2 vs. 3-6 and mRS 0-4 vs. 5-6, boxplots will be shown for TAI versus outcome, for treatment and control arm patients separately. In addition, probability of the outcome variable, calculated from the ORs will be plotted: TAI on the x-axis and *probability* of the outcome of interest on the y-axis. This will be done for adjusted/unadjusted results, and treatment subgroups. Confidence intervals will be plotted around the probability lines. For post-EVT reperfusion, boxplots will be shown for TAI versus reperfusion, for IV rtPA treatment yes and no separately. For final infarct volume, the linear regression results will be shown in a scatter plot with TAI on the x-axis and final infarct volume on the y-axis, for adjusted/unadjusted results, and treatment subgroups.

PRISMA-IPD Checklist of items to include when reporting a systematic review and meta-analysis of individual participant data (IPD)

PRISMA-IPD Section/topic	Item No	Checklist item	Reported on page
**Title**			
Title	1	Identify the report as a systematic review and meta-analysis of individual participant data.	4
**Abstract**			
Structured summary	2	Provide a structured summary including as applicable:	2
		**Background**: state research question and main objectives, with information on participants, interventions, comparators and outcomes.	2
		**Methods**: report eligibility criteria; data sources including dates of last bibliographic search or elicitation, noting that IPD were sought; methods of assessing risk of bias.	2
		**Results**: provide number and type of studies and participants identified and number (%) obtained; summary effect estimates for main outcomes (benefits and harms) with confidence intervals and measures of statistical heterogeneity. Describe the direction and size of summary effects in terms meaningful to those who would put findings into practice.	2
		**Discussion:** state main strengths and limitations of the evidence, general interpretation of the results and any important implications.	3
		**Other:** report primary funding source, registration number and registry name for the systematic review and IPD meta-analysis.	-
**Introduction**			
Rationale	3	Describe the rationale for the review in the context of what is already known.	3
Objectives	4	Provide an explicit statement of the questions being addressed with reference, as applicable, to participants, interventions, comparisons, outcomes and study design (PICOS). Include any hypotheses that relate to particular types of participant-level subgroups.	4
**Methods**			
Protocol and registration	5	Indicate if a protocol exists and where it can be accessed. If available, provide registration information including registration number and registry name. Provide publication details, if applicable.	4
Eligibility criteria	6	Specify inclusion and exclusion criteria including those relating to participants, interventions, comparisons, outcomes, study design and characteristics (e.g. years when conducted, required minimum follow-up). Note whether these were applied at the study or individual level i.e. whether eligible participants were included (and ineligible participants excluded)	4

		from a study that included a wider population than specified by the review inclusion criteria. The rationale for criteria should be stated.	
Identifying studies - information sources	7	Describe all methods of identifying published and unpublished studies including, as applicable: which bibliographic databases were searched with dates of coverage; details of any hand searching including of conference proceedings; use of study registers and agency or company databases; contact with the original research team and experts in the field; open adverts and surveys. Give the date of last search or elicitation.	4, HERMES original pooling report: **Goyal et al. Lancet 2016**[1]
Identifying studies - search	8	Present the full electronic search strategy for at least one database, including any limits used, such that it could be repeated.	N/A
Study selection processes	9	State the process for determining which studies were eligible for inclusion.	4
Data collection processes	10	Describe how IPD were requested, collected and managed, including any processes for querying and confirming data with investigators. If IPD were not sought from any eligible study, the reason for this should be stated (for each such study).<br><br>If applicable, describe how any studies for which IPD were not available were dealt with. This should include whether, how and what aggregate data were sought or extracted from study reports and publications (such as extracting data independently in duplicate) and any processes for obtaining and confirming these data with investigators.	4, HERMES original pooling report[1]
Data items	11	Describe how the information and variables to be collected were chosen. List and define all study level and participant level data that were sought, including baseline and follow-up information. If applicable, describe methods of standardising or translating variables within the IPD datasets to ensure common scales or measurements across studies.	4, HERMES original pooling report[1]
IPD integrity	A1	Describe what aspects of IPD were subject to data checking (such as sequence generation, data consistency and completeness, baseline imbalance) and how this was done.	4, HERMES original pooling report[1]
Risk of bias assessment in individual studies.	12	Describe methods used to assess risk of bias in the individual studies and whether this was applied separately for each outcome. If applicable, describe how findings of IPD checking were used to inform the assessment. Report if and how risk of bias assessment was used in any data synthesis.	4, HERMES original pooling report[1]
Specification of outcomes and effect measures	13	State all treatment comparisons of interests. State all outcomes addressed and define them in detail. State whether they were pre-specified for the review and, if applicable, whether they were primary/main or secondary/additional outcomes. Give the principal measures of effect (such as risk ratio, hazard ratio, difference in means) used for each outcome.	5
Synthesis methods	14	Describe the meta-analysis methods used to synthesise IPD. Specify any statistical methods and models used. Issues should include (but are not restricted to):<br><br>• Use of a one-stage or two-stage approach.	4-7

		• How effect estimates were generated separately within each study and combined across studies (where applicable).<br>• Specification of one-stage models (where applicable) including how clustering of patients within studies was accounted for.<br>• Use of fixed or random effects models and any other model assumptions, such as proportional hazards.<br>• How (summary) survival curves were generated (where applicable).<br>• Methods for quantifying statistical heterogeneity (such as I^2 and τ^2).<br>• How studies providing IPD and not providing IPD were analysed together (where applicable).<br>• How missing data within the IPD were dealt with (where applicable).	
Exploration of variation in effects	A2	If applicable, describe any methods used to explore variation in effects by study or participant level characteristics (such as estimation of interactions between effect and covariates). State all participant-level characteristics that were analysed as potential effect modifiers, and whether these were pre-specified.	6
Risk of bias across studies	15	Specify any assessment of risk of bias relating to the accumulated body of evidence, including any pertaining to not obtaining IPD for particular studies, outcomes or other variables.	4, HERMES original pooling report[1]
Additional analyses	16	Describe methods of any additional analyses, including sensitivity analyses. State which of these were pre-specified.	6
**Results**			
Study selection and IPD obtained	17	Give numbers of studies screened, assessed for eligibility, and included in the systematic review with reasons for exclusions at each stage. Indicate the number of studies and participants for which IPD were sought and for which IPD were obtained. For those studies where IPD were not available, give the numbers of studies and participants for which aggregate data were available. Report reasons for non-availability of IPD. Include a flow diagram.	7, Supplementary Methods: PRISMA-flowchart
Study characteristics	18	For each study, present information on key study and participant characteristics (such as description of interventions, numbers of participants, demographic data, unavailability of outcomes, funding source, and if applicable duration of follow-up). Provide (main) citations for each study. Where applicable, also report similar study characteristics for any studies not providing IPD.	4, HERMES original pooling report[1]
IPD integrity	A3	Report any important issues identified in checking IPD or state that there were none.	4, HERMES original pooling report[1]
Risk of bias within studies	19	Present data on risk of bias assessments. If applicable, describe whether data checking led to the up-weighting or down-weighting of these assessments. Consider how any potential bias impacts on the robustness of meta-analysis conclusions.	HERMES original pooling report[1]
Results of individual studies	20	For each comparison and for each main outcome (benefit or harm), for each individual study report the number of eligible participants for which data were obtained and show simple summary data for each intervention group (including, where	10, HERMES original pooling report[1] and original trials[2–8]

		applicable, the number of events), effect estimates and confidence intervals. These may be tabulated or included on a forest plot.	Individual reporting of substudy results per trial is not possible under the HERMES pooling agreements.
Results of syntheses	21	Present summary effects for each meta-analysis undertaken, including confidence intervals and measures of statistical heterogeneity. State whether the analysis was pre-specified, and report the numbers of studies and participants and, where applicable, the number of events on which it is based.	7-10
		When exploring variation in effects due to patient or study characteristics, present summary interaction estimates for each characteristic examined, including confidence intervals and measures of statistical heterogeneity. State whether the analysis was pre-specified. State whether any interaction is consistent across trials.	10
		Provide a description of the direction and size of effect in terms meaningful to those who would put findings into practice.	7-12
Risk of bias across studies	22	Present results of any assessment of risk of bias relating to the accumulated body of evidence, including any pertaining to the availability and representativeness of available studies, outcomes or other variables.	4, HERMES original pooling report[1]
Additional analyses	23	Give results of any additional analyses (e.g. sensitivity analyses). If applicable, this should also include any analyses that incorporate aggregate data for studies that do not have IPD. If applicable, summarise the main meta-analysis results following the inclusion or exclusion of studies for which IPD were not available.	10-12
**Discussion**			
Summary of evidence	24	Summarise the main findings, including the strength of evidence for each main outcome.	12-13
Strengths and limitations	25	Discuss any important strengths and limitations of the evidence including the benefits of access to IPD and any limitations arising from IPD that were not available.	14
Conclusions	26	Provide a general interpretation of the findings in the context of other evidence.	13-14
Implications	A4	Consider relevance to key groups (such as policy makers, service providers and service users). Consider implications for future research.	16
**Funding**			
Funding	27	Describe sources of funding and other support (such as supply of IPD), and the role in the systematic review of those providing such support.	16

A1 – A3 denote new items that are additional to standard PRISMA items. A4 has been created as a result of re-arranging content of the standard PRISMA statement to suit the way that systematic review IPD meta-analyses are reported.

© Reproduced with permission of the PRISMA IPD Group, which encourages sharing and reuse for non-commercial purpose

1. Goyal M, Menon BK, Van Zwam WH, Dippel DWJ, Mitchell PJ, Demchuk AM, Dávalos A, Majoie CBLM, Van Der Lugt A, De Miquel MA, et al. Endovascular thrombectomy after large-vessel ischaemic stroke: A meta-analysis of individual patient data from five randomised trials. *Lancet*. 2016;387:1723–1731.

2. O.A. Berkhemer, P.S.S. Fransen, D. Beumer, L.A. van den Berg, H.F. Lingsma, A.J. Yoo, W.J. Schonewille, J.A. Vos, P.J. Nederkoorn, M.J.H. Wermer, M.A.A. van Walderveen, J. Staals, J. Hofmeijer, J.A. van Oostayen, G.J. Lycklama à Nijeholt, J. Boiten, P.A. RH Lo, E.J. van Dijk, J. de Vries, P.L.M. de Kort, W.J.J. van Rooij, J.S.P. van den Berg, B.A.A.M. van Hasselt, L.A.M. Aerden, R.J. Dallinga, M.C. Visser, J.C.J. Bot, P.C. Vroomen, O. Eshghi, T.H.C.M.L. Schreuder, R.J.J. Heijboer, K. Keizer, A.V. Tielbeek, H.M. PJK, W.H. van Zwam, Y.B.W.E.M. Roos, A. van der Lugt, R.J. van Oostenbrugge, C.B.L.M. Majoie, and D.W.J. Dippel for the MCI. A Randomized Trial of Intraarterial Treatment for Acute Ischemic Stroke. *N. Engl. J. Med.* 2015;372:11–20.

3. Goyal M, Demchuk AM, Menon BK, Eesa M, Rempel JL, Thornton J, Roy D, Jovin TG, Willinsky RA, Sapkota BL, et al. Randomized assessment of rapid endovascular treatment of ischemic stroke. *N Engl J Med* [Internet]. 2015;372:1019–1030. Available from: http://www.ncbi.nlm.nih.gov/pubmed/25671798

4. Campbell BCV, Mitchell PJ, Churilov L, Yassi N, Kleinig TJ, Dowling RJ, Yan B, Bush SJ, Dewey HM, Thijs V, et al. Tenecteplase versus Alteplase before Thrombectomy for Ischemic Stroke. *N. Engl. J. Med.* 2018;378:1573–1582.

5. Saver JL, Goyal M, Bonafe A, Diener H-C, Levy EI, Pereira VM, Albers GW, Cognard C, Cohen DJ, Hacke W, et al. Stent-Retriever Thrombectomy after Intravenous t-PA vs. t-PA Alone in Stroke. *N. Engl. J. Med.* [Internet]. 2015;372:2285–2295. Available from: http://www.ncbi.nlm.nih.gov/pubmed/25882376%5Cnhttp://www.nejm.org/doi/abs/10.1056/NEJMoa1415061

6. Bracard S, Ducrocq X, Mas JL, Soudant M, Oppenheim C, Moulin T, Guillemin F. Mechanical thrombectomy after intravenous alteplase versus alteplase alone after stroke (THRACE): a randomised controlled trial. *Lancet Neurol.* [Internet]. 2016;15:1138–1147. Available from: http://dx.doi.org/10.1016/S1474-4422(16)30177-6

7. Muir KW, Ford GA, Messow C-M, Ford I, Murray A, Clifton A, Brown MM, Madigan J, Lenthall R, Robertson F, et al. Endovascular therapy for acute ischaemic stroke: the Pragmatic Ischaemic Stroke Thrombectomy Evaluation (PISTE) randomised, controlled trial. *J. Neurol. Neurosurg. Psychiatry* [Internet]. 2016;jnnp-2016-314117. Available from: http://www.ncbi.nlm.nih.gov/pubmed/27756804

8. Jovin TG, Chamorro A, Cobo E, de Miquel MA, Molina CA, Rovira A, Román LS, Serena J, Abilleira S, Ribó M, et al. Thrombectomy within 8 Hours after Symptom Onset in Ischemic Stroke. *N. Engl. J. Med.* [Internet]. 2015;372:1–11. Available from: http://www.ncbi.nlm.nih.gov/pubmed/25882510

PRISMA IPD Flow Diagram

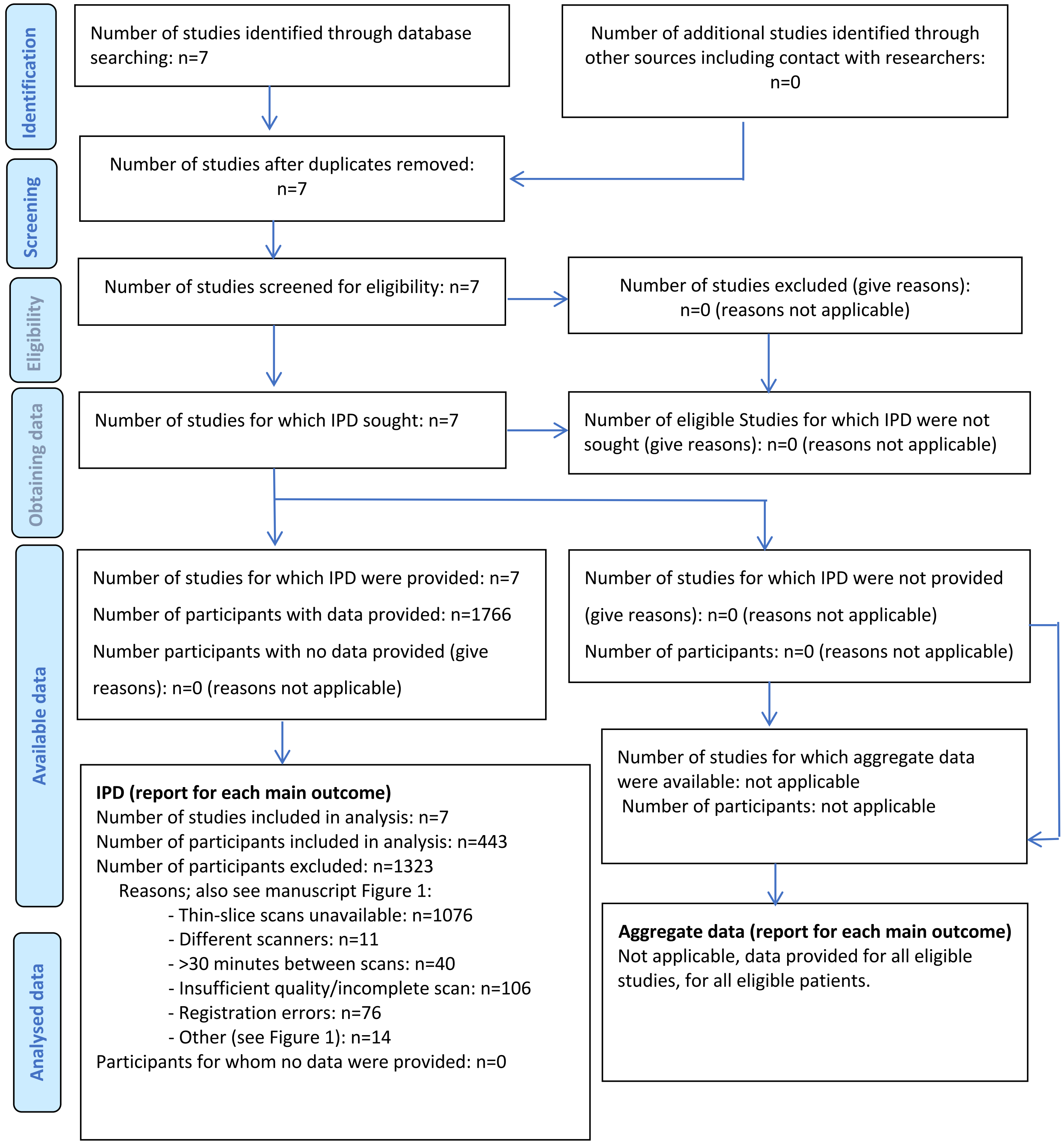

The PRISMA IPD flow diagram

© Reproduced with permission of the PRISMA IPD Group, which encourages sharing and reuse for non commercial purposes

SUPPLEMENTAL RESULTS

Imaging acquisition characteristics

Most scans were acquired with a scanner tube voltage of 120 kVp (median 120, IQR 120-120 for NCCT; 110-120 for CTA). Median NCCT slice thickness was 1.0 mm (IQR 0.6-1.0), median CTA slice thickness was 0.8 mm (IQR 0.6-1.0). Most scans were acquired on Philips scanners (170/443, 38%).

Effect of scan acquisition characteristics on thrombus perviousness

Slice thickness difference between NCCT and CTA varied significantly between TAI quartiles (p<0.01; **Supplemental Results Figure A**). Larger slice thickness difference (larger NCCT slices versus thinner CTA slices) corresponded to larger TAI. TAI values and thrombus density on NCCT differed significantly between scanner brands (p<0.01; **Supplemental Results Figure C and F**). Thrombus density on NCCT decreased with increasing NCCT slice thickness (τ=-0.08, p=0.02; **Supplemental Results Figure D**). No significant effect of slice thickness or scanner brand on clot length was observed (p=0.73, p=0.41 respectively). No effect of peak kilovoltage (kVp) on TAI or NCCT thrombus density was observed (**Supplemental Results Figure B and E**).

**Imaging acquisition characteristics**		**Current cohort (N=443)**		*Known in*
NCCT scanner tube voltage (kVp) – *median (IQR)*		120	*(120-120)*	*443*
CTA scanner tube voltage (kVp) – *median (IQR)*		120	*(110-120)*	*443*
NCCT slice thickness (mm) – *median (IQR)*		1.0	*(0.6-1.0)*	*443*
CTA slice thickness (mm) – *median (IQR)*		0.8	*(0.6-1.0)*	*443*
Scanner brand – *N (%)*	Siemens	170	*(38%)*	*443*
	GE Medical Systems	131	*(30%)*	*443*
	Philips	96	*(22%)*	*443*
	Toshiba	44	*(10%)*	*443*
	PNMS	2	*(0.5%)*	*443*

Supplemental Results Table 1. Imaging acquisition characteristics. Median (interquartile range) for continuous variables. Number (%) for categorical variables. *CTA, CT angiography; GE, general electric; IQR, interquartile range; kVp, peak kilovoltage; NCCT, non-contrast CT; PNMS, Philips-Neusoft Medical Systems.*

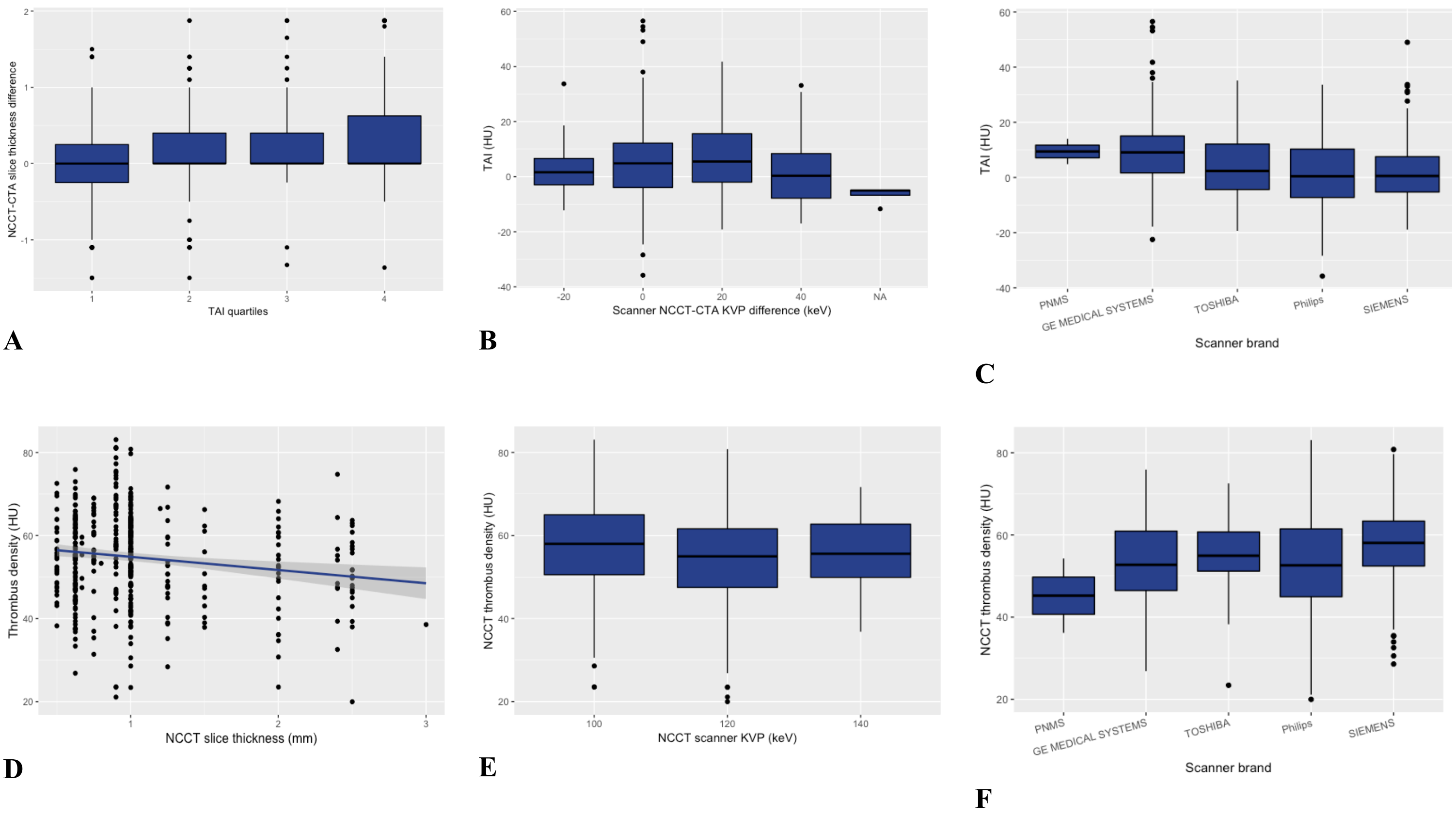

Supplemental Results Figure A-F. Thrombus characteristics and acquisition details.
A, NCCT-CTA slice thickness difference for every quartile of TAI. **B**, TAI versus NCCT-CTA scanner kVp difference. **C**, TAI versus scanner brands. D, NCCT thrombus density versus slice thickness. **E**, NCCT thrombus density versus scanner kVp. **F**, NCCT thrombus density versus scanner brand. *TAI, thrombus attenuation increase; HU, Hounsfield Units; NCCT, non-contrast CT; CTA, CT angiography.*

SUPPLEMENTAL TABLES

**Clinical characteristics (total N=443)**		**1st TAI quartile (n=111)**	**2nd TAI quartile (n=110)**	**3rd TAI quartile (n=111)**	**4th TAI quartile (n=111)**	*p=*
Age (yr) [total N]	*– median (min-max)*	67 *(35-93)* [110]	70 *(39-88)* [110]	70 *(39-88)* [111]	68 *(29-90)* [111]	0.22
Male sex – *n/total N (%)*		66/110 (60%)	63/110 *(57%)*	50/111 *(45%)*	57/111 *(51%)*	0.12
Left hemisphere stroke – *n/total N (%)*		51/105 (49%)	46/99 *(47%)*	57/93 *(61%)*	44/92 *(48%)*	0.15
Baseline NIHSS – *median (IQR)*		18 *(5-38)*	18 *(4-32)*	18 *(4-31)*	17 *(5-30)*	0.29
Treatment allocation – *n/total N (%)*	Intervention	55/110 *(50%)*	52/110 *(47%)*	54/111 *(49%)*	49/111 *(44%)*	0.84
	Control	55/110 *(50%)*	58/110 *(53%)*	57/111 *(51%)*	62/111 *(56%)*	
IV-alteplase – *N/total N (%)*	Intervention	83.6% (46/55)	80.8% (42/52)	87.0% (47/54)	89.8% (44/49)	0.61
	Control	90.9% (50/55)	94.8% (55/58)	87.7% (50/57)	90.3% (56/62)	
Time onset-randomization (min) – *median (IQR)*		212 *(55-471)*	195 *(59-448)*	175 *(73-551)*	185 *(50-708)*	0.27
Atrial fibrillation – *n/total N (%)*		36/107 *(34%)*	29/103 *(28%)*	26/97 *(27%)*	30/95 *(32%)*	0.70
Diabetes mellitus – *n/total N (%)*		20/110 *(18%)*	20/110 *(18%)*	16/111 *(14%)*	18/111 *(16%)*	0.85
Hypertension – *n/total N (%)*		57/110 *(52%)*	61/110 *(56%)*	56/111 *(51%)*	64/111 *(58%)*	0.69
**Imaging characteristics**						
TAI (HU)	*– median (IQR)*	-8 *(-12—10)*	0 *(-2-1)*	7 *(5-9)*	18 *(14-26)*	*NA*
	– mean (± SD)	-10 *(±5)*	0 *(±2)*	7 *(±2)*	21 *(±10)*	*NA*
Occlusion location – *n/total N (%)*	ICA	26 *(23%)*	26 *(24%)*	27 *(23%)*	13 *(12%)*	*0.09*
	M1	80 *(72%)*	79 *(72%)*	77 *(69%)*	95 *(86%)*	
	M2	4 *(4%)*	5 *(5%)*	7 *(6%)*	3 *(3%)*	
	Other (A2)	1 *(1%)*	0 *(0%)*	0 *(0%)*	0 *(0%)*	
NCCT thrombus density (HU) – *median (IQR)*		61 *(54-66)*	57 *(51-62)*	54 *(48-62)*	50 *(41-58)*	***<0.01****
Thrombus length (mm) – *median (IQR)*		18 *(12-25)*	17 *(12-22)*	16 *(10-23)*	14 *(8-22)*	***<0.01****
NCCT scanner tube voltage (kVp) – *median (IQR)*		120 *(100-120)*	120 *(120-120)*	120 *(120-120)*	120 *(120-120)*	***<0.01****

CTA scanner tube voltage (kVp) – *median (IQR)*		120 *(100-120)*	120 *(100-120)*	120 *(120-120)*	120 *(120-120)*	***<0.01****
NCCT slice thickness (mm) – *median (IQR)*		0.9 *(0.8-1.0)*	1.0 *(0.8-1.0)*	1.0 *(0.6-1.0)*	0.9 *(0.6-1.4)*	***0.02****
CTA slice thickness (mm) – *median (IQR)*		0.9 *(0.6-1.0)*	0.8 *(0.6-1.0)*	0.6 *(0.6-0.9)*	0.6 *(0.6-0.9)*	***<0.01****
Scanner brand – *n/total N (%)*	Siemens	50 *(45%)*	53 *(48%)*	43 *(38%)*	24 *(22%)*	***<0.01****
	GE Medical Systems	15 *(14%)*	21 *(19%)*	43 *(39%)*	52 *(47%)*	
	Philips	34 *(31%)*	25 *(23%)*	16 *(14%)*	21 *(19%)*	
	Toshiba	12 *(11%)*	11 *(10%)*	8 *(7%)*	13 *(12%)*	
	PNMS	0 *(0%)*	0 *(0%)*	1 *(1%)*	1 *(1%)*	
**Follow-up**						
90-day mRS– *n/total N (%)*	0	3/110 *(3%)*	8/109 *(7%)*	9/109 *(8%)*	7/110 *(6%)*	***<0.01****
	1	11/110 *(10%)*	14/109 *(13%)*	12/109 *(11%)*	14/110 *(13%)*	
	2	10/110 *(9%)*	11/109 *(10%)*	23/109 *(21%)*	24/110 *(22%)*	
	3	17/110 *(16%)*	26/109 *(24%)*	23/109 *(21%)*	25/110 *(23%)*	
	4	34/110 *(31%)*	19/109 *(17%)*	20/109 *(18%)*	16/110 *(15%)*	
	5	8/110 *(7%)*	9/109 *(8%)*	9/109 *(8%)*	12/110 *(11%)*	
Mortality (mRS 6) – *n/total N (%)*		27/110 *(25%)*	22/110 *(20%)*	13/109 *(12%)*	12/110 *(11%)*	***0.02****
Post-EVT reperfusion – *n/total N (%)*	eTICI 2b-3	35/48 *(73%)*	34/47 *(72%)*	36/44 *(82%)*	29/41 *(71%)*	0.63
sICH – *N/total N (%)*		7/109 *(6%)*	6/110 *(6%)*	8/110 *(7%)*	1/110 *(1%)*	0.13
Final infarct volume (ml) – *median (IQR)*		85 *(28-193)*	64 *(21-156)*	46 *(16-107)*	32 *(12-97)*	***<0.01****

Supplemental Table I. Baseline and follow-up characteristics per quartile of TAI. Median (interquartile range) for continuous variables. Number (%) for categorical variables. *A1, segment one of anterior cerebral artery; IQR, interquartile range; SD, standard deviation; IV-alteplase, intravenous tissue plasminogen activator; EVT, endovascular treatment; TAI, thrombus attenuation increase; ICA, internal carotid artery; ICA-T, ICA terminus; kVp, peak kilovoltage; M1, segment one of medial cerebral artery; M2, segment two of medical cerebral artery; mRS, modified Rankin scale score; eTICI, extended thrombolysis in cerebral infarction score; N, number of patients; NA, not applicable/available; sICH, symptomatic intracranial hemorrhage; yr, years. * Indicates statistical significance.*

**Outcome measure**	**uOR** ***(95% CI)***	**Interaction with EVT**	**uOR per arm** ***(95% CI)***
Ordinal mRS	**1.13** ***(1.05-1.20)****	***p*****=0.04***	Control: **1.23** ***(1.12-1.34)********
			Intervention: 1.03 *(0.92-1.15)*
mRS 0-2	**1.17** ***(1.07-1.27)****	*p*=0.07	*NA*
mRS 5-6	**0.91** ***(0.83-0.99)****	*p*=0.02	Control: **0.82** ***(0.73-0.94)****
			Intervention: 1.02 *(0.90-1.17)*
Mortality	**0.84** ***(0.75-0.93)****	*p*=0.50	*NA*
Final infarct volume (effect ratio)	**0.90** ***(0.85-0.94)****	*p*=0.10$	Control: **0.86** ***(0.81-0.92)****
			Intervention: 0.95 *(0.88-1.03)*
Intervention arm only: Successful reperfusion	0.99 *(0.84-1.18)*	*Interaction with IV alteplase:* *p*=0.34	*NA*

Supplemental Table II. Unadjusted odds ratios (uOR, aOR) for the effect of increased TAI (per 5 HU) on outcomes. Control arm: intravenous alteplase if eligible (n=232), intervention arm: additional EVT (n=210). *EVT, endovascular treatment; TAI, thrombus attenuation increase; mRS, modified Rankin scale score; NA, not applicable/available.*

[#] *Adjusted for: age (years), baseline NIHSS, IV-alteplase yes/no, occlusion location, diabetes mellitus, stroke onset to randomization time, NCCT-CTA slice thickness difference; and including random effects for study and scanner brand.*

** Indicates statistical significance.*

[$] *Exploratory analysis in model with non-significant treatment interaction.*

Outcome measure	uOR *(95% CI)*	Interaction with EVT	uOR per arm *(95% CI)*	aOR# *(95% CI)*	Interaction with EVT	aOR# per arm *(95% CI)*
Ordinal mRS	**1.15** ***(1.07-1.23)****	***p*=0.05***	Control: **1.25 *(1.13-1.36)****	**1.10 *(1.03-1.18)****	***p*=0.03***	Control: **1.22 *(1.11-1.35)****
			Intervention: 1.04 *(0.93-1.17)*			Intervention: 0.98 *(0.87-1.11)*

Supplemental Table III. Exploratory analysis in intravenous alteplase-treated patients only (N=390; 179 intervention arm, 211 control arm). Unadjusted odds ratios (uOR) and adjusted odds ratios (aOR) for the effect of increased TAI (analyzed per 5 HU) on each outcome measure. Control arm consisted of intravenous alteplase if eligible (n=232), intervention arm consisted of additional EVT (n=210). *EVT, endovascular treatment; TAI, thrombus attenuation increase; mRS, modified Rankin scale score.*
# Adjusted for: age (years), NIHSS at baseline, IV-alteplase yes/no, occlusion location, diabetes mellitus, stroke onset to randomization time, NCCT-CTA slice thickness difference; and including random effects for study and scanner brand.
** Indicates statistical significance.*

	**ucOR *(95% CI)* for EVT** Intervention arm versus control arm	**acOR# *(95% CI)* for EVT** Intervention arm versus control arm
Outcome: ordinal mRS		
TAI Q1 (n=111)	**2.21 *(1.11-4.31)****	**2.85 *(1.29-6.30)****
TAI Q2 (n=110)	**2.95 *(1.47-5.95)****	**3.68 *(1.73-7.83)****
TAI Q3 (n=111)	**2.33 *(1.17-4.65)****	**2.06 *(1.00-4.24)****
TAI Q4 (n=111)	0.86 *(0.44-1.71)*	1.01 *(0.49-2.08)*

Supplemental Table IV. Exploratory analysis of EVT effect per quartile of TAI, on the primary outcome (ordinal mRS). Unadjusted odds ratios (uOR) and adjusted odds ratios (aOR) for the effect of increased TAI (analyzed per 5 HU). Control arm consisted of intravenous alteplase if eligible (n=232), intervention arm consisted of additional EVT (n=210). *EVT, endovascular treatment; TAI, thrombus attenuation increase; mRS, modified Rankin scale score.*
# Adjusted for: age (years), NIHSS at baseline, IV-alteplase yes/no, occlusion location, diabetes mellitus, stroke onset to randomization time, NCCT-CTA slice thickness difference; and including random effects for study and scanner brand.
** Indicates statistical significance.*

SUPPLEMENTAL FIGURES

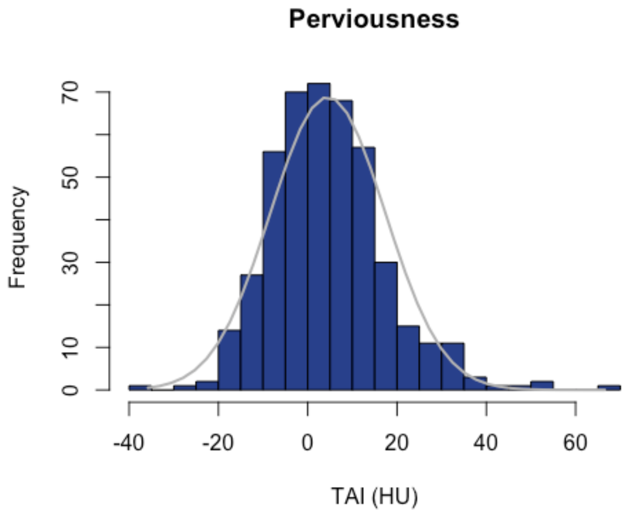

Supplemental Figure I. TAI distribution. *TAI, thrombus attenuation increase; HU, Hounsfield Units*

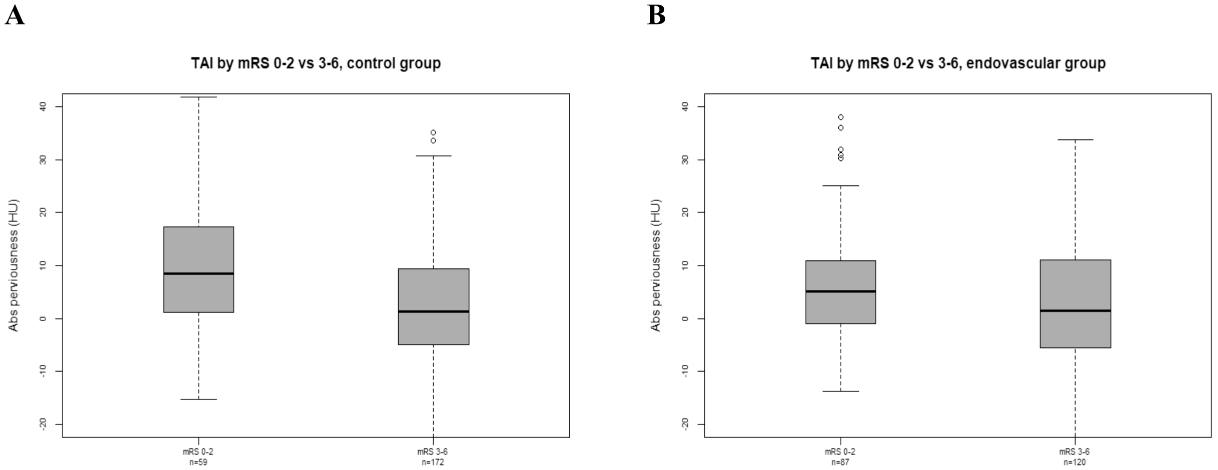

Supplemental Figure II. TAI for mRS 0-2 (left boxes) and 3-6 (right boxes) patients. A, control and **B**, intervention arm. *TAI, thrombus attenuation increase; mRS, modified Rankin Scale; HU, Hounsfield Units; abs perviousness, perviousness; thrombus attenuation increase.*

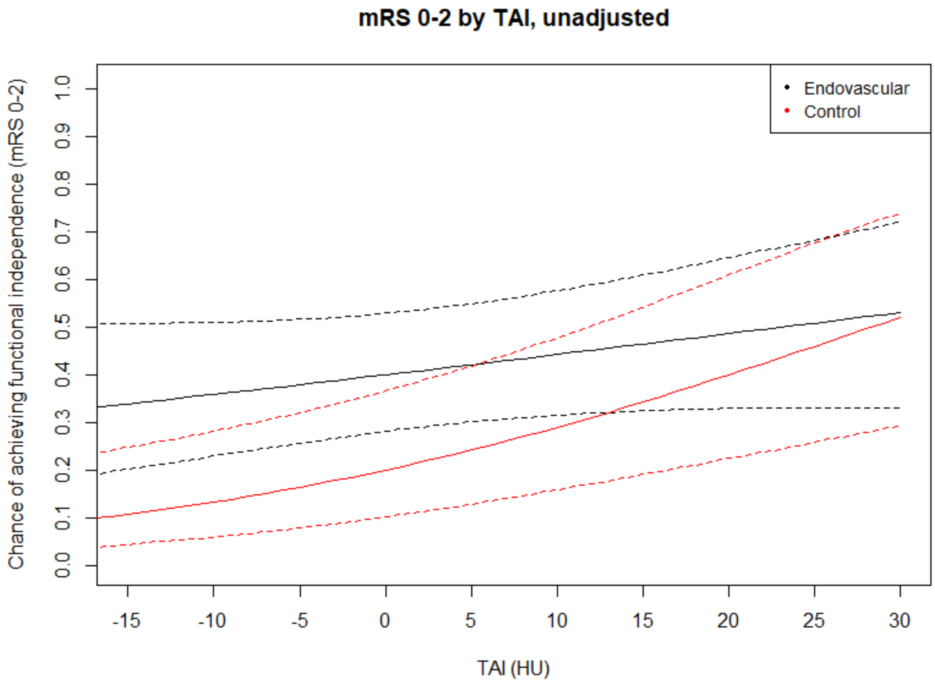

Supplemental Figure III. Unadjusted probability of functional independence (mRS 0-2 at 90 days), versus TAI. *TAI, thrombus attenuation increase; HU, Hounsfield Units; mRS, modified Rankin scale.* Control arm consisted of standard care (IV recombinant tissue plasminogen activator if eligible). Intervention arm consisted of endovascular treatment in addition to standard care.

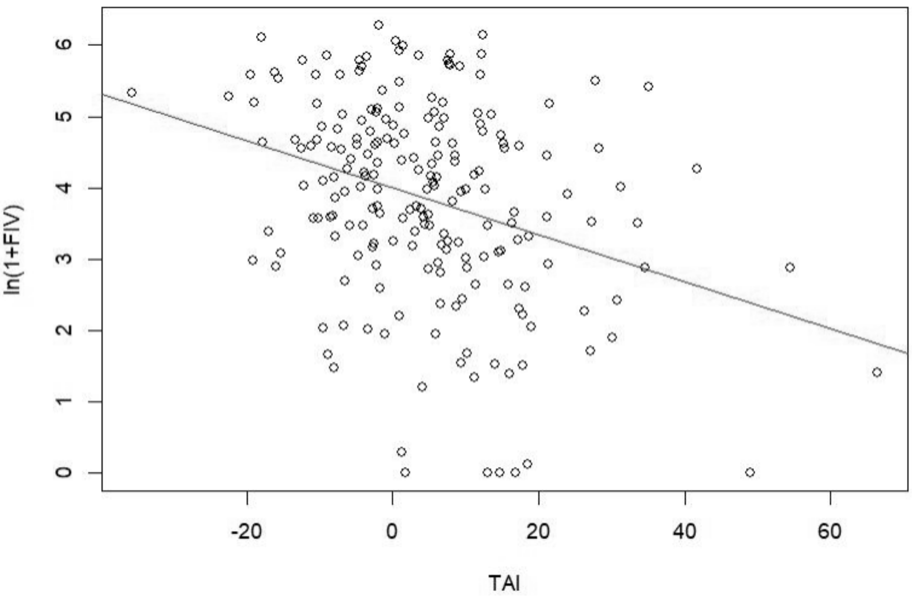

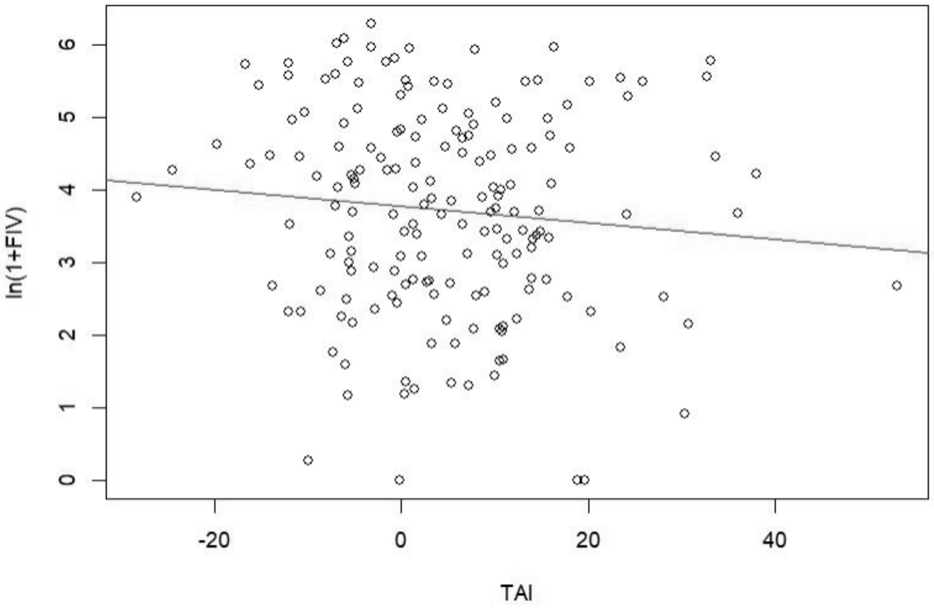

Supplemental Figure IV. Scatter plots of final infarct volume versus TAI, for control (upper) and intervention arm (lower). *Transformation by ln(1+FIV) was performed because of a right-skewed distribution of FIV. FIV, final infarct volume; TAI, thrombus attenuation increase.*
