# Supplementary figures and images for: Endovascular Treatment Effect Diminishes With Increasing Thrombus Perviousness: Pooled Data From 7 Trials on Acute Ischemic Stroke
Source: Stroke. 2021 Jul 20;52(11):3633–41. doi: 10.1161/STROKEAHA.120.033124 (PMC8547583; doi:10.1161/STROKEAHA.120.033124)

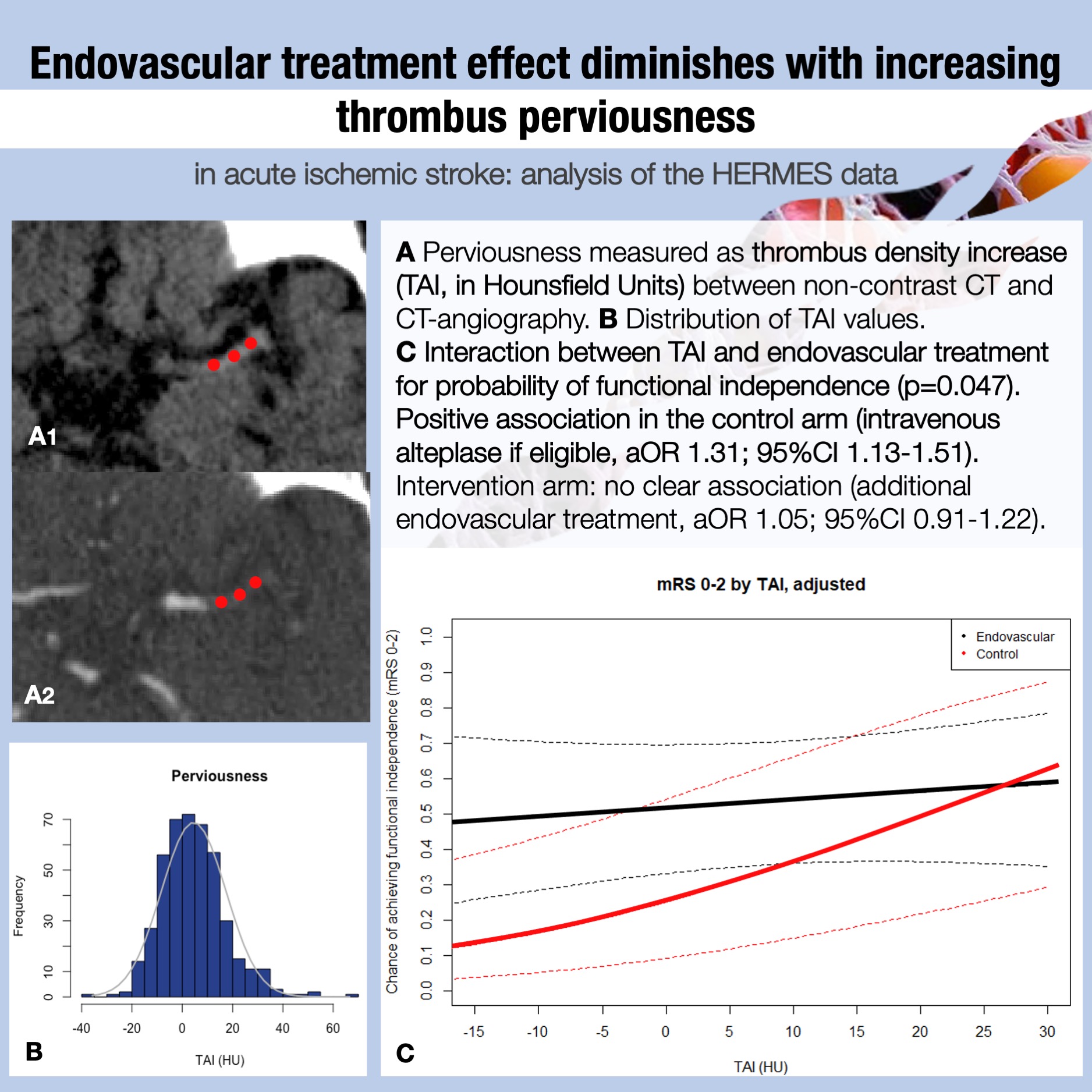

Supplement: Supplementary file 2 [file str-52-3633-s002.jpg]
